# Genetic and DNA Methylation Changes in Cotton (*Gossypium*) Genotypes and Tissues

**DOI:** 10.1371/journal.pone.0086049

**Published:** 2014-01-20

**Authors:** Kenji Osabe, Jenny D. Clement, Frank Bedon, Filomena A. Pettolino, Lisa Ziolkowski, Danny J. Llewellyn, E. Jean Finnegan, Iain W. Wilson

**Affiliations:** CSIRO, Plant Industry, ACT, Australia; Nanjing Agricultural University, China

## Abstract

In plants, epigenetic regulation is important in normal development and in modulating some agronomic traits. The potential contribution of DNA methylation mediated gene regulation to phenotypic diversity and development in cotton was investigated between cotton genotypes and various tissues. DNA methylation diversity, genetic diversity, and changes in methylation context were investigated using methylation-sensitive amplified polymorphism (MSAP) assays including a methylation insensitive enzyme (*Bsi*SI), and the total DNA methylation level was measured by high-performance liquid chromatography (HPLC). DNA methylation diversity was greater than the genetic diversity in the selected cotton genotypes and significantly different levels of DNA methylation were identified between tissues, including fibre. The higher DNA methylation diversity (CHG methylation being more diverse than CG methylation) in cotton genotypes suggest epigenetic regulation may be important for cotton, and the change in DNA methylation between fibre and other tissues hints that some genes may be epigenetically regulated for fibre development. The novel approach using *Bsi*SI allowed direct comparison between genetic and epigenetic diversity, and also measured CC methylation level that cannot be detected by conventional MSAP.

## Introduction

Cytosine methylation is a flexible epigenetic regulatory mechanism that controls gene expression by inhibiting proteins binding to DNA and by changing the structure of the associated chromatin. In plants, DNA methylation can occur on cytosines in any context (CG, CHG and asymmetric CHH, where H is A, C or T) with CG being the most commonly methylated dinucleotide [1], [2]. CG and non-CG methylation can silence transposons and pseudogenes, and regulate plant development and tissue specific gene expression [3], [4]. CG, CHG and CHH methylation are established through *de novo* methylation dependent on small RNAs, but maintained through different processes [5]. In *Arabidopsis*, CG methylation is maintained by METHYLTRANSFERASE1(MET1), CHG methylation is maintained by DOMAINS REARRANGED METHYLTRANSFERASE1/2 (DRM1/2) and CHROMOMETHYLASE3 (CMT3), while DECREASE in DNA METHYLATION1 (DDM1) is required for both CG and non-CG methylation [6]–[10].

Changes in DNA methylation levels between developmental stages or tissues can indicate the involvement of epigenetic regulation. The total DNA methylation level measured by Methylation-sensitive amplified polymorphism (MSAP) in different tissues or developmental stages in maize [11], rice [12], sorghum [13], or Arabidopsis [14] is around 16–40%. Generally, the methylation level increases as the tissue matures [14], [15], and endosperm tissue is often hypomethylated [16]–[19]. In cotton, changes in the levels of DNA methylation between cotyledon, seedling leaf, mature plant leaf, and roots were observed [20]–[22], but the relative level of DNA methylation in cotton fibre compared to other tissues is not known. Changes in DNA methylation level between developmental stages or tissues suggest that epigenetic regulation may be important in creating phenotypic diversity.

The requirement for DNA methylation in plant development has been demonstrated by the pleiotropic phenotypes observed when the epigenome was disrupted by down-regulating genes such as *DDM1* and *MET1* that are required for DNA methylation [10], [23], [24], or by chemical treatment [25], [26]. Loss of DNA methylation can influence plant traits such as yield, fruit ripening, seed size, flowering time, plant size, plant stature, sex determination, and pathogen resistance [10], [27]–[32]. In cotton, there have been reports of DNA methylation changes related to response to light quality, heterosis, salt-tolerance, alkali stress, and annual habit [20]–[22], [33]–[35].

Epigenetic changes can occur more frequently than spontaneous genetic mutations [36], [37], allowing phenotypic plasticity and divergence. Higher DNA methylation diversity compared to the genetic diversity has been reported previously in *Viola cazorlensis* [38] and *Brassica oleracea* [39], which suggests the potential involvement of epigenetic regulation of phenotypic traits. Cotton has limited genetic diversity [40]–[43] due to a relatively recent polyploidization event [44] and subsequent domestication, but the extent of DNA methylation polymorphism is greater compared to the genetic polymorphisms in *G. hirsutum* accessions collected from different geographical regions around the world [45]. DNA methylation may be contributing to increased phenotypic diversity in cotton, including in fibre traits that have been selected during domestication and breeding.

Cotton fibres, which are widely used for textile production, are elongated single cell seed trichomes growing from the epidermis of the outer integument of ovules. Cotton ovules have two layers of integuments, outer and inner integuments, which develop into the seed coat after fertilisation. About 30% of the epidermal cells in the outer integument form fibre initials [46], which expand, elongate, and thicken over about 50 days post anthesis (dpa) to form mature fibres. *G. hirsutum* L. and *G. barbadense* are the most common cultivated cotton species. *G. hirsutum* dominates global cotton production being grown for its high fibre yield, whereas *G. barbadense* is grown for its high fibre quality. Both species are allotetraploids (AADD-genome) derived from diploid progenitors similar to present day G. *arboreum* (A-genome) and *G. raimondii* (D-genome) species, and have superior fibre yield and quality relative to their ancestors [44], [47]. Complex genomic and epigenomic change affecting gene expression is thought to accompany polyploid formation [48]–[51], and this is likely to have contributed to the improved fibre traits of the polyploids. Many fibre-related genes change expression during fibre development [52], but it is not known whether these genes are epigenetically regulated.

To understand the potential contribution of DNA methylation regulation to the phenotypic diversity and plant development in cotton, the change in DNA methylation between cotton genotypes and various tissues was investigated. The DNA methylation diversity and genetic diversity was compared using methylation-sensitive amplified polymorphism (MSAP) assays and the total methylation level was measured by high-performance liquid chromatography (HPLC). MSAP analyses demonstrated higher DNA methylation diversity than genetic diversity in the selected cotton genotypes, and significantly different levels of DNA methylation were observed between tissues of the *G. hirsutum* cultivar Coker 315-11.

## Materials and Methods

### Plant Materials

*Gossypium hirsutum* L. genotypes Namcala, Delta Pine 16 (DP16), Sicot 75, Sicot 71, Coker 315-11 and three advanced breeding lines CSX6280, CSX5150, and CSX4184, and *G. barbadense* genotypes Sipima 280 and CPX12 were used for genotype comparisons. The genotypes were selected to represent a range of short/long fibre length and weak/strong fibre strength (Fig. S1). *G. barbadense* genotypes were used as a genetic outlier for our analysis. Pedigree analysis (data not shown) shows Namcala as the most distant genotype to other *G. hirsutum* genotypes. DP16 and Coker 315 are closely related, and Sicot 71, Sicot 75, CSX6280, CSX5150, and CSX4184 form a separate group.

Field experiments were grown at the Australian Cotton Research Institute (ACRI) near Narrabri, NSW, Australia (30°S; 150°E). The soil type was heavy grey clay, Vertosol classified as Ug5.2 [53]. Genotypes were planted in three rows by 12 meter plots with three replications. Field experiments were sown in early October 2010 in rows 100 cm apart. Crops were managed with full irrigation, spraying for insect pests as required and weeds controlled by pre-planting application of herbicides such as trifluralin and Fluorometuron followed by inter-row cultivation prior to flowering.

Cotton leaf samples were taken in February 2011 with the crop being near the cutout stage of development. Five fully expanded leaves from twenty individual plants were sampled from the inner row of each plot. The samples were placed immediately in liquid nitrogen, and transferred to −80°C for storage. Care was taken to select leaves at a similar developmental stage to minimize potential epigenetic variability. After harvesting, cotton was ginned on a 20 saw laboratory gin, and fibre quality was analysed with a High Volume Instrument (HVI; Uster technologies Inc., Charlotte, NC) for fibre length and strength.

A separate glasshouse experiment was performed from July to September 2011 for tissue analyses in Coker 315 grown at Canberra, ACT, Australia. Three-week-old plants were used to collect cotyledon, stems and total root tissues, while 6-month-old mature plants were used to collect fully expanded (mature) leaves, primary roots, 0 dpa and 3 dpa ovules, and 35 dpa fibre (manually separated from seeds). The outer integument (OI) and inner integument (II) were dissected from 0 dpa ovules harvested between 13∶00–15∶00 [54]. Two flowers were combined as one replicate for OI and II, and for each tissue, three or four biological replicates were collected from individual plants.

### DNA Extraction

All DNA extraction was performed using modified a DNeasy mini plant DNA extraction kit (Qiagen, Melbourne, Australia). DNA extraction for genotype comparisons was performed by adding polyvinylpyrrolidone (20 mgml^−1^) to Buffer AP1, and followed the manufacturer’s instructions. DNA extraction from tissues was performed by adding polyvinylpyrrolidone (20 mgml^−1^) to buffer AP1, and including a 10 minute incubation with 10 mM dithiothreitol, and 0.5 mgml^−1^ Proteinase K (final concentration) at 65°C, after the RNase A incubation step, and followed the manufacturer’s instructions. DNA quality was determined by the 260/280 ratio from the Nanodrop spectrophotometer (Thermo Scientific, Melbourne, Australia) and visual integrity of the DNA bands by gel electrophoresis in 1.2% TAE agarose.

### HPLC

DNA digest for quantifying methylcytosine was performed as previously described [55], and the digested DNA was separated using a reverse-phase HPLC [56] with modifications. Modifications were made on the HPLC run as follows: Hold at methanol/50 mM KH2PO4 [2.5/97.5](v/v) for 5 min, linear gradient to methanol/KH2PO4 [25/75] over 8 min, and linear gradient back to methanol/KH2PO4 [2.5/97.5] over 2 min. HPLC System Gold (Beckman Coulter, Sydney, Australia) fitted with ZORBAX Eclipse XBD-C18 column, 4.6×150 mm, 5-micron (Agilent, Sydney, Australia) was used for DNA separation, and absorbance at 280 nm (and reference absorbance at 320 nm) was measured by a diode array detector.

Standards containing cytidine, uridine, guanosine, adenosine, 2′-deoxy cytidine, 5-methyl-2′-deoxycytidine, thymidine, deoxy-guanosine, deoxy-adenosine at concentration of 1 µg/ml each, were used to determine retention times of each nucleoside. The average area under the peak were standardised to six 2-fold dilution series of the standards, and the area of deoxy cytidine (dC) and methyl dC was used to calculate the percentage of methyl dC (%mdC) to the amount of total cytosine (methyl dC+dC). Oligonucleotides (5′-TCGAATTCGGCCATGGCCGAATTCGA-3′) containing 0%, 28.5%, and 57.1% methylcytosine (substituted at two or four C’s with methyldeoxycytosine) were synthesized (Sigma, Sydney, Australia) and used as controls for DNA digest and methylcytosine quantification. Peaks were analysed using System Gold (Beckman Coulter, Sydney, Australia) and Microsoft Excel, and error rates were calculated for DNA extraction, digest, and HPLC runs (Table S1).

### MSAP

MSAP was performed for each genotype in three biological replicates [57], with modifications. Modification made were: 500 ng of template DNA, double-digest using combinations of *Eco*RI and *Bsi*SI (Jenabioscience, Jena, Germany), *Hpa*II (New England Biolabs, Arundel, Australia) or *Msp*I (New England Biolabs, Gold Coast, Australia), PCR using FastStart Taq polymerase (Roche diagnostics, Sydney, Australia), fluorescently labelled reactions (FAM, VIC, NED, PET) were mixed, and peaks were separated using an ABI3130×l capillary sequencer (Applied Biosystems, Melbourne, Australia). Traces were analysed using GeneMarker software (SoftGenetics, Pennsylvania, USA). Adaptors and oligonucleotides used are listed in Table S2. The pre-amplification and selective amplification cycling conditions were performed as manufacturer’s instruction with 40 cycles for the selective amplification.

Scoring of each CCGG site was automated to assess the presence (“1”) or absence (“0”) of peaks using GeneMarker. A panel for each oligonucleotide pair was constructed based on all methylation insensitive (*Eco*RI/*Bsi*SI) data of *G. hirsutum* and *G. barbadense* genotypes. The panel was manually refined by selecting the peaks that were strong and consistent in at least two of the three replicates. The panel constructed using methylation insensitive data was applied to all genotype/tissue samples to produce binary data for genetic analysis. To minimize biological and technical scoring errors that occur between each replicate, a consensus score was constructed for each site, producing a single binary data point for each genotype/tissue. All three enzyme combinations recognize the same CCGG site, hence the panels constructed from the methylation insensitive data was applied to the methylation sensitive EcoRI/*Hpa*II and *Eco*RI/*Msp*I data to automate binary data for DNA methylation analysis. The inclusion of *Bsi*SI is a novel modification of the conventional MSAP method that allowed analysis of additional sites that could not otherwise be assessed, and to directly compare the genetic diversity to the DNA methylation diversity at the same CCGG site.

#### MSAP data analysis

A total of 28 oligonucleotide pairs were used to generate 389 bands that could be scored reliably across the tissues, and 44 primer pairs were used to generate 1120 bands that could be scored reliably across the ten genotypes, with 1084 bands (subtracting the *G. barbadense* specific bands) from just the *G. hirsutum* genotypes. The polymorphism ratio within each *Eco*RI/*Bsi*SI data set was determined by calculating the total number of polymorphic sites within the genotypes divided by the total number of sites analysed. The percentage polymorphism of the methylation sensitive enzyme was calculated by total number of DNA methylation polymorphic sites identified, divided by the total number of sites analysed. The calculated DNA methylation polymorphic sites do not exclude sites that are polymorphic both genetically and by DNA methylation.

DNA methylation level was quantified for each genotype and tissue using the MSAP binary data. Presence of peaks in the *Eco*RI/*Msp*I and absence in *Eco*RI/*Hpa*II was considered CG methylated site, presence of peaks in *Eco*RI/*Hpa*II and absence in *Eco*RI/*Msp*I was considered CHG methylation site, and absence of peaks in both were considered CC methylation (Table S3). However, when CHG is methylated on both strands *Hpa*II and *Msp*I cannot cleave the site, and is represented in the CC methylation (i.e. CC is inclusive of double-strand CHG methylation). The CG, CHG, and CC methylation level was calculated by the number of absent peaks in *Eco*RI/*Hpa*II or *Eco*RI/*Msp*I divided by the total number of peaks analysed in the *Eco*RI*/Bsi*SI data. Genetically polymorphic sites were excluded in the analysis.

The similarity coefficient (Simple matching), cluster analysis, Mantel’s test, and the principal component analysis (PCA) were performed using NTSYS v2.2 [58]. Simple matching method considers the double-absence of peaks as additional information in a pair-wise comparison for closely related species [59]. This is also appropriate for assessing the *G. hirsutum* genotypes as these genotypes are expected to have low heterozygosity, and the presence/absence of the bands are likely due to homology rather than homoplasy (different DNA fragments from different ancestral origin comigrating). Use of Jaccard and Dice coefficients [60], [61] for closely related species is commonly used when it is not known whether the double-absence of peaks in pair-wise comparison are due to DNA sequence polymorphism or homoplasy [62]. However, in this study, the methylation insensitive (*Eco*RI/*Bsi*SI) data had bands present across most genotypes and showed that the absence of peaks were not due to sequence polymorphism, and the potential contribution of homoplasy is expected to be very small. Absence of bands in pair-wise comparison of genotypes in both *Eco*RI/*Hpa*II and *Eco*RI/*Msp*I data indicates that this region is likely to share the same methylation state. Unweighted pair group method with arithmetic mean (UPGMA) dendrogram was constructed using the simple matching similarity coefficient and DendroUPGMA [63]. Principal component analysis (PCA) was performed using a correlation matrix of the polymorphic fragments of the genotypes as previously described [45], and used to visualize the relatedness of the genotypes using the “PCA batch module” of NTSYS v2.21 to represent the relatedness between each sample by its spacial distance.

### Statistical Analysis

Significant differences between each sample’s methylation level determined by HPLC or MSAP were analysed by One-way ANOVA and Tukey’s test using BrightStat [64]. The dendrograms generated from MSAP were supported by Mantel’s test with 1000 permutations (Table S4) and Bootstrap test with 2000 permutations (95% accuracy) [65] using NTSYS v2.21 and Winboot [66], respectively. The average error rate per locus was calculated for each tissue/genotype and each MSAP enzyme combination [67] and are included in Table S4.

### Comparison of Fibre Phenotypic Diversity to MSAP Diversity

The Euclidean distance of fibre length and strengths was calculated using NTSYS v2.21 as a measure of distance between each genotype. The computed Euclidean distance matrix and the dissimilarity matrix computed from the simple matching correlation coefficient were assessed for correlation using Mantel’s test with 1000 permutations (Table S5).

## Results

### DNA Methylation Analysis of Tissues

**Figure 1:**
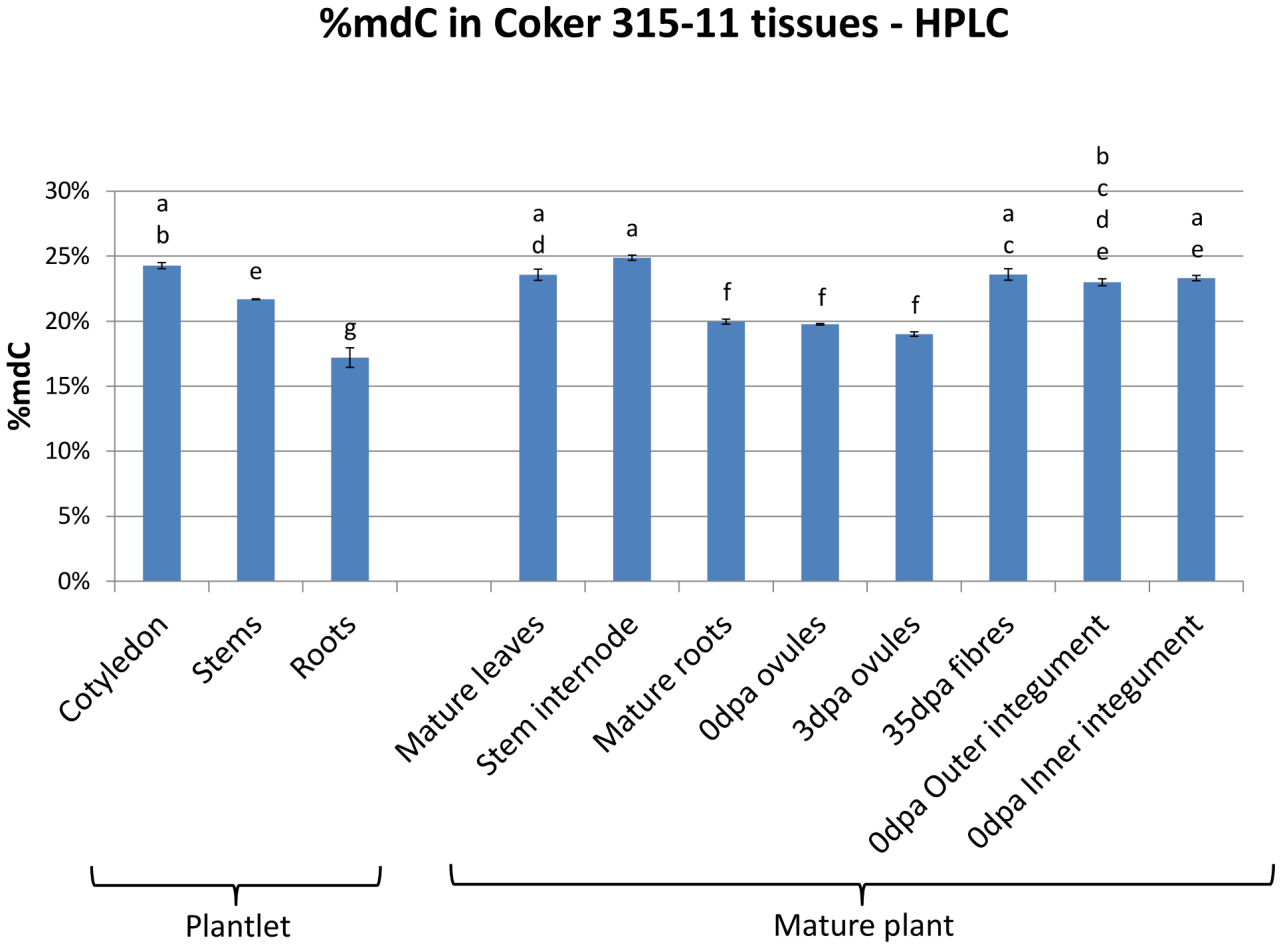
Percentage of total methylation (%mdC) level of Coker 315-11 tissues determined by HPLC. Different letters above the bars denotes samples that have significantly different levels of methylation (p-value of <0.05). The error bars represent the standard error of the mean. Different tissues from 3-week-old plantlet and 6-months-old mature plant were used.

HPLC and MSAP assays were used to monitor DNA methylation level and context in different tissues of cotton that were grown in the glasshouse. DNA methylation level of tissues harvested from 3-week-old plantlet (cotyledon, stems, roots) and 6-month-old mature plant (mature leaves, stem internode, mature roots, 0 dpa ovules, 3 dpa ovules, 35 dpa fibres, outer integument and inner integument of 0 dpa ovules) were assessed by HPLC (Figure 1). The 0 dpa ovules, 3 dpa ovules, and 35 dpa fibres represent fibre initiation, early elongation, and late elongation stage [47], [68], respectively. Both inner integument and outer integument are derived from the ovule primordium, and the inner integument develops more slowly and independently of the outer integument [69], [70]. The outer integument gives rise to fibre initials (visible at 0 dpa) that develop into fibres and the remaining epidermal cells form the epidermis of the seed coat. The separation of fibre initials from surrounding epidermal cells is difficult, and recently, a method has been developed to isolate the outer integument of ovules that enriches for cells (∼30% of epidermal cells) that produce fibre [54]. The comparison of the fibre forming cells on the outer integument to the non-fibre forming inner integument may be useful to understand the changes during fibre development.

Plantlet roots had the lowest percentage of total methylation (17%mdC) and cotyledon, stem internodes, mature leaves, 35 dpa fibre, and inner and outer integument had the highest methylation (∼23–25%mdC). DNA methylation levels in plantlet cotyledon, stems and roots were significantly different from each other, and the level of methylation in cotyledons was comparable to that of mature leaves and stem internodes of mature plants. Plantlet roots had lower methylation (17%mdC) than mature roots (20%mdC). Both 0 dpa ovules and 3 dpa ovules possessed significantly lower methylation (20%mdC) than the outer and inner integument harvested from ovules at 0 dpa (23%mdC). The 35 dpa fibre (∼23%mdC) was comparable to 0 dpa outer and inner integument.

**Figure 2:**
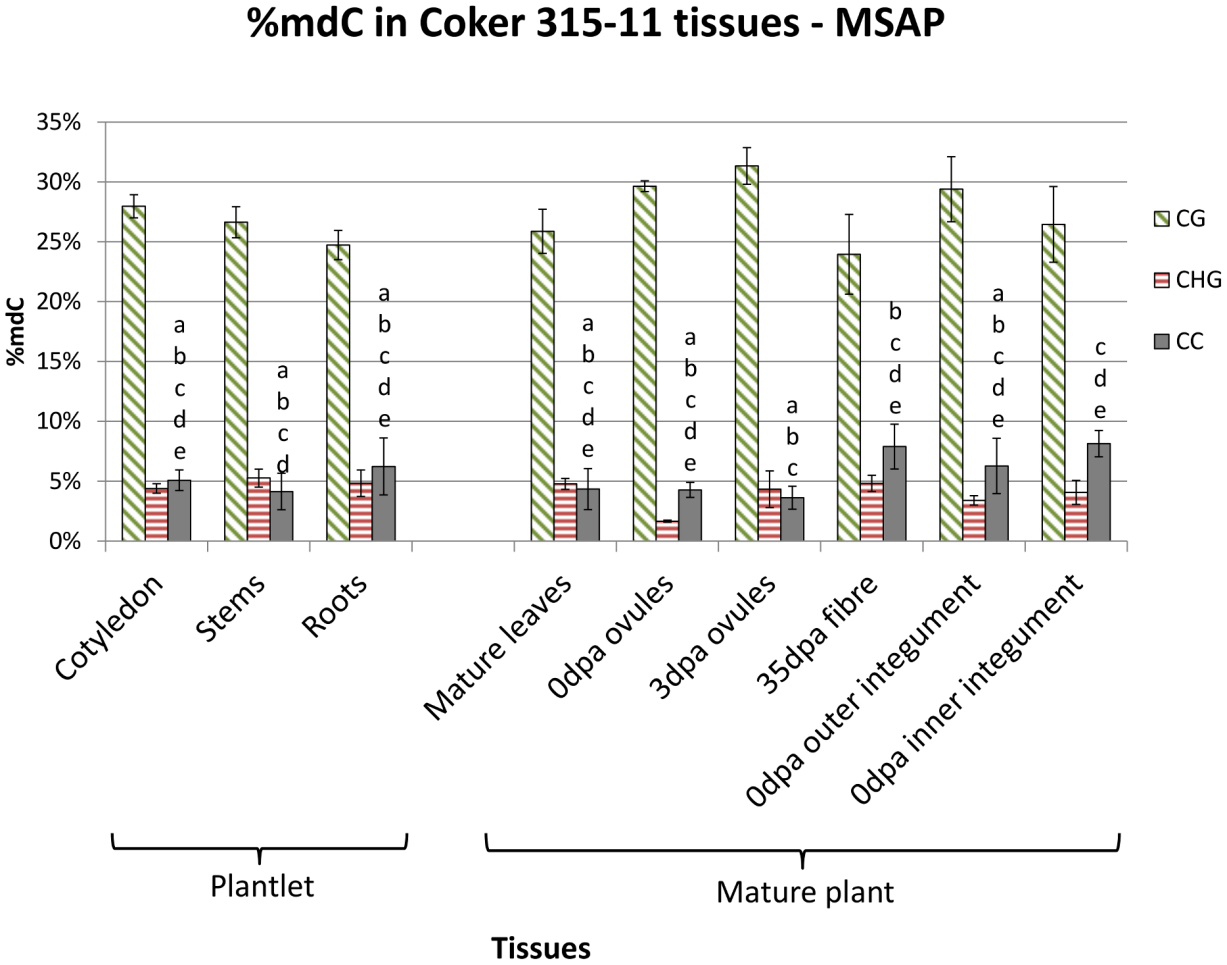
Methylation level of Coker 315-11 tissues determined by MSAP. Methylation was categorized into CG, CHG and CC methylation and represented as a bar graph. Significant differences are denoted by different letters for CC methylation. The error bars represent the standard error of mean from at least three biological replicates. No significant differences for CG and CHG methylation were found.

A subset of tissues used in HPLC analysis was selected for MSAP analysis to investigate the methylation context in fibre-developing tissues relative to other tissues (Figure 2). MSAP assays examine the context of DNA methylation using the methylation-sensitive isoschizomers *Hpa*II and *Msp*I, allowing discrimination between CG and CHG methylation. The addition of *BsiSI* (a methylation insensitive isoschizomer of *Hpa*II and *Msp*I) permitted the identification CC methylation of the CCGG sites that were not cleaved by either *Hpa*II or *Msp*I. Methylation was divided into three categories, scored as CG, CHG and CC methylation according to MSAP data (Table S3). Significant differences were found only in the CC methylation context (Figure 2). Lowest CC methylation was found in 3 dpa ovules and the highest were 35 dpa fibre and 0 dpa inner integument.

To visualise the relationship of CG and CHG methylation between different tissues, the MSAP data were used to construct a dendrogram (Fig. S2). The overall relationship between the tissues for CG and CHG was similar, indicating a potential relationship in the CG and CHG methylation pattern in different tissues.

The number of methylation polymorphic sites was determined for outer integument, inner integument, and 35 dpa fibre to identify any DNA methylation changes that occur between these tissues during fibre development. Using *Bsi*SI, 389 fragments were detected; of these 28 CG (7.2%) and 23 CHG (5.9%) methylation polymorphisms were found between outer and inner integuments. There were 55 CG (14.1%) and 56 CHG (14.4%) methylation polymorphisms between outer integument and 35 dpa fibre fragments, and fibre had the most number of unique polymorphisms amongst the three tissues (Fig S3).

### DNA Methylation Comparison between Genotypes

#### HPLC

**Figure 3:**
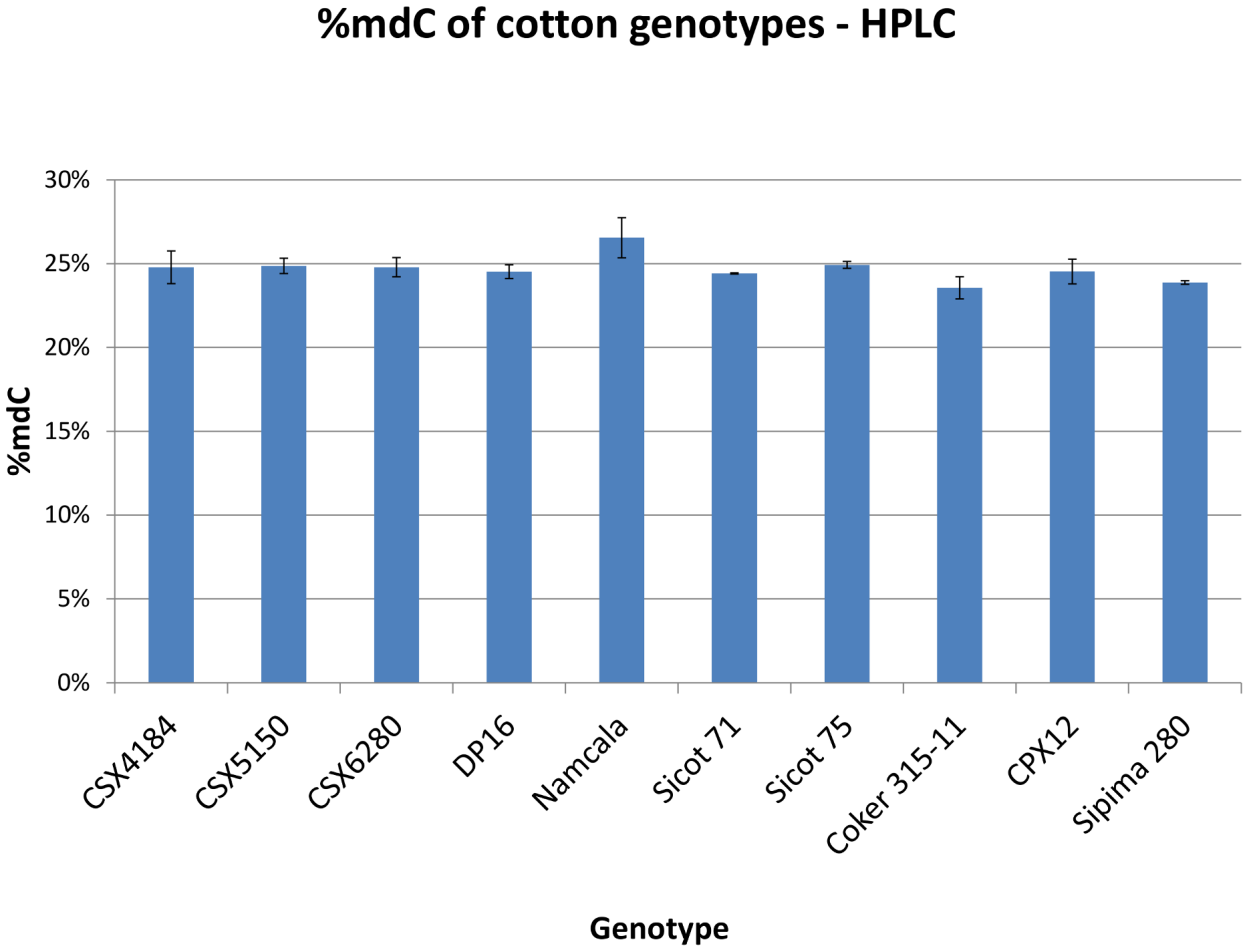
Methylation level of leaves for each genotype quantified by HPLC. The average global methylation level measured by HPLC for *G.* hirsutum and *G.* barbadense were 24.8% and 24.2%, respectively. The error bars represent the standard error of mean calculated from two biological replicates. No significant differences were identified between genotypes.

The methylation level of DNA for two biological replicates was measured by HPLC for each cotton genotype (Figure 3). There were no significant differences of %mdC between genotypes within *G. hirsutum,* and the average methylation level between species was very similar (24.8% and 24.2%) for *G. hirsutum* and *G. barbadense*, respectively.

#### Level of DNA methylation by MSAP (CG, CHG, CC)

The ten genotypes, including both *G. hirsutum* and *G. barbadense*, were selected to represent diversity of fibre length and strength (Fig. S1). The changes in DNA methylation context between the ten genotypes were measured by MSAP to compare the genetic diversity and fibre quality diversity. The use of *Bsi*SI allowed a direct comparison between genetic and DNA methylation diversity of the selected cotton genotypes.

**Figure 4:**
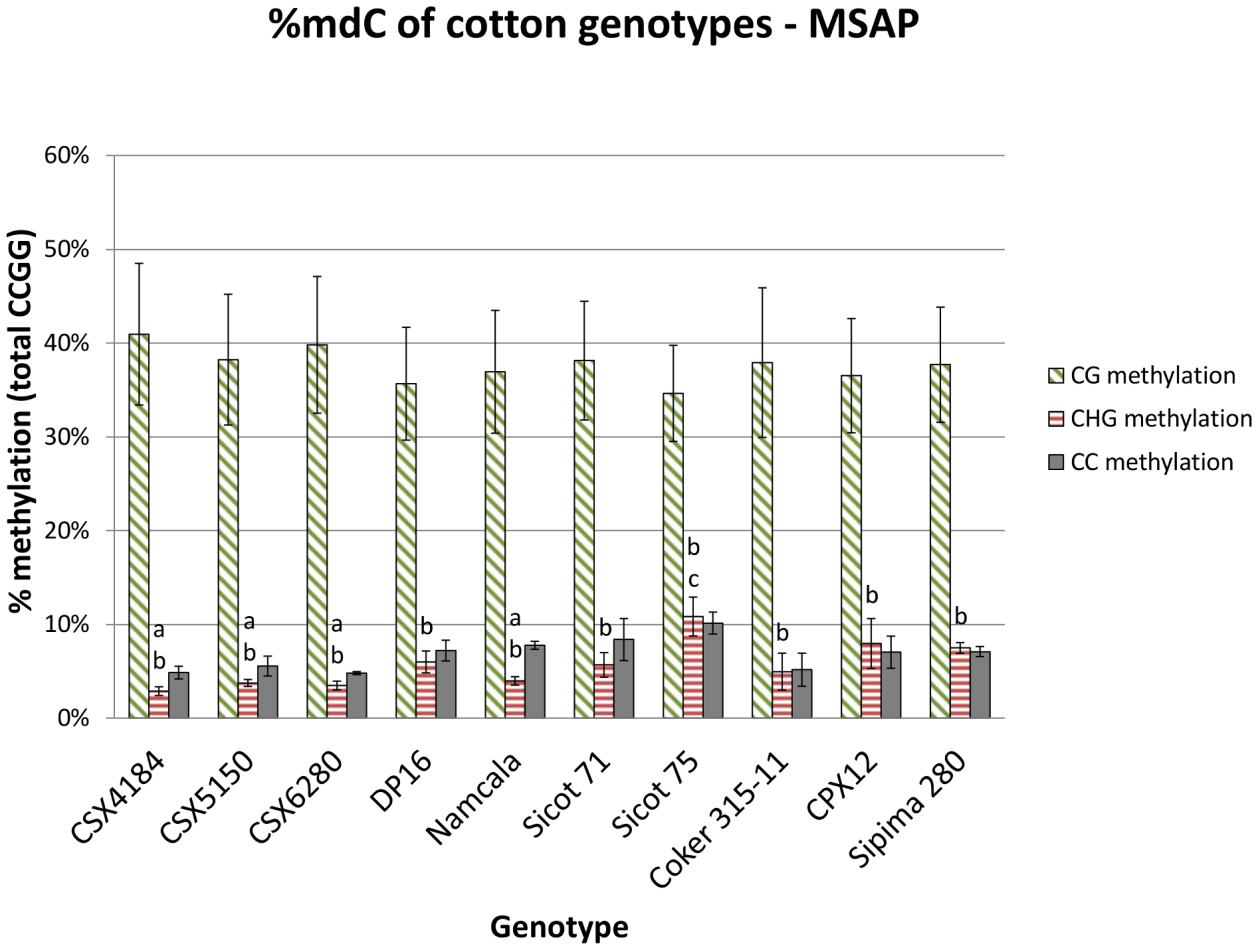
Methylation level of cotton genotypes determined by MSAP. The average genome-wide methylation level measured by MSAP for *G. hirsutum* and *G. barbadense* was 49.7% and 51.9%, respectively. No significant differences in CG or CC methylation were found between genotypes. Significant differences found in CHG methylation is denoted by different letters.

The extent of methylation at CCGG sites was determined using MSAP data (Figure 4). The average CG, CHG, and CC methylation level of the eight *G. hirsutum* genotypes were 37.8%, 5.2%, and 6.7% (49.7% total methylation), respectively. The average CG, CHG, and CC methylation level for the two *G. barbadense* genotypes was comparable to that of *G. hirsutum*. Within *G. hirsutum*, the total methylation of CCGG sites assessed was 7.5–8 percentage points different between Sicot 75 and CSX5150/CSX6280 (p<0.05). The amount of CHG methylation differed between Sicot 75 and CSX4184/CSX5150/CSX6280/Namcala (p<0.05), but no significant differences were observed across *G. hirsutum* and *G. barbadense*.

#### Diversity analysis by Simple Matching (SM) coefficient

The proportion of polymorphic sites (number of polymorphic sites within the total number of assessed bands) for all ten genotypes and within *G. hirsutum* genotypes were calculated for each of the enzyme combinations (Table S6) Within *G. hirsutum* genotypes, the number of CHG polymorphisms was about 1.5-fold and CG polymorphism was 3-fold more than the genetic polymorphism. The similarity coefficient determined using the simple matching method for the ten genotypes averaged 0.878 (range = 0.729–0.976) for *Eco*RI/*Bsi*SI, 0.877 (range = 0.797–0.937) for *Eco*RI/*Hpa*II, and 0.837 (range = 0.709–0.945) for *Eco*RI/*Msp*I. Considering the *G. hirsutum* genotypes only, the similarity coefficient averaged 0.932 (range = 0.85–0.975) for *Eco*RI/*Bsi*SI, 0.9 (range = 0.834–0.935) for *Eco*RI/*Hpa*II, and 0.88 (range = from 0.768–0.945) for *Eco*RI/*Msp*I. As expected, *G. barbadense* genotypes were genetically more distant than any of the *G. hirsutum* genotypes. Irrespective of whether they were compared across all genotypes or within *G. hirsutum*, the genetic diversity was very low and the DNA methylation diversity was greater than the genetic diversity. Comparing CG and CHG methylation, the CHG methylation was more diverse for both species.

**Figure 5:**
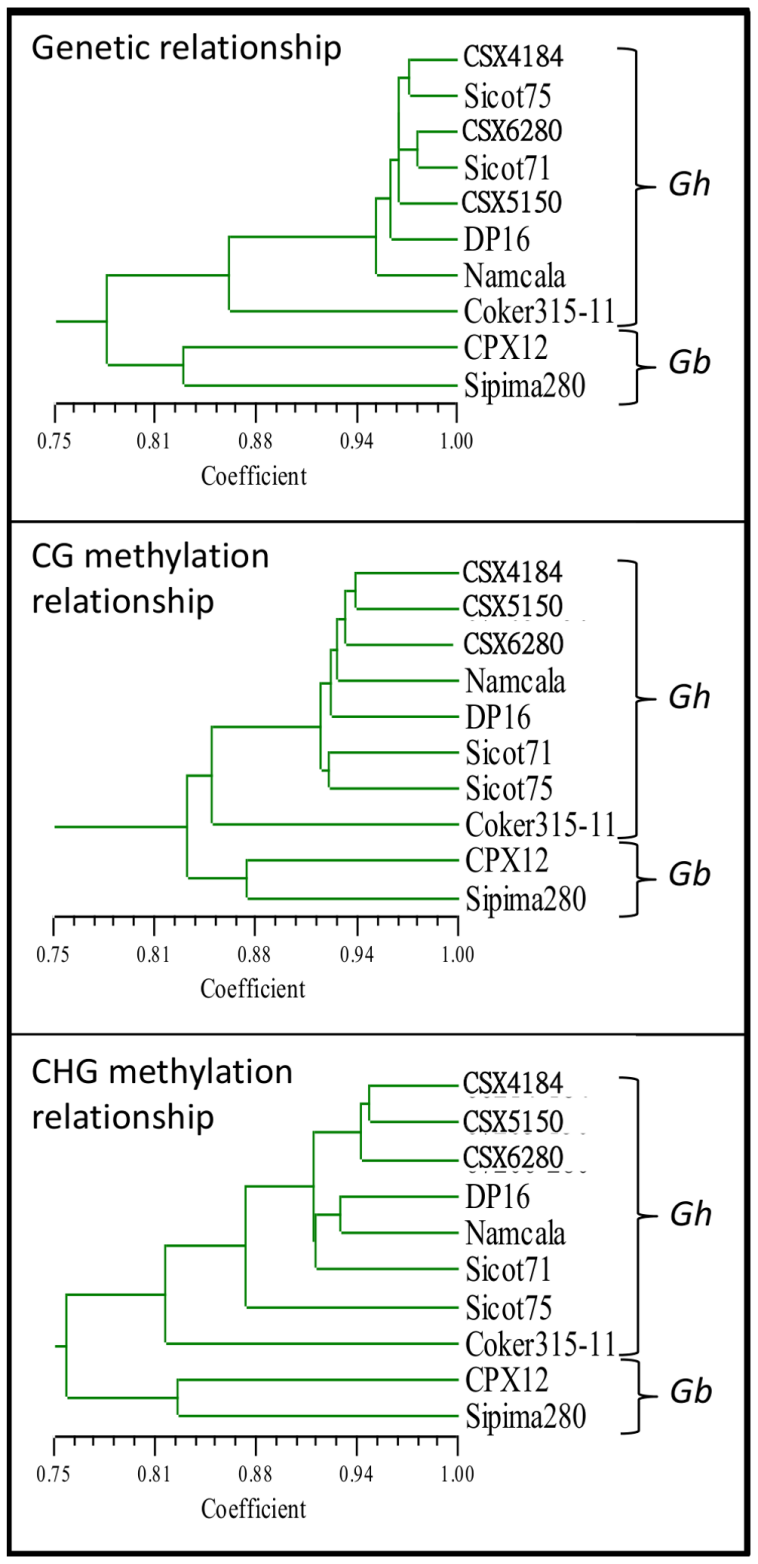
Dendrogram of the ten genotypes constructed using the similarity coefficient. *G. hirsutum* species are indicated by “*Gh*” and *G. barbadense* species are indicated by “*Gb*”. The separation of species and Coker 315-11 from other *G. hirsutum* genotypes is consistent in all dendrograms, but the relationships between G. hirsutum genotypes are different. Separation of genetic, CHG and CG methylation clusters in *G. hirsutum* genotypes (except Coker 315-11) show clear genetic/DNA methylation divergence in cultivated cotton.

Simple matching similarity coefficients were used to construct dendrograms to represent the relationship between genotypes and their DNA methylation state (Figure 5). Genotypes methylation analysis formed four clades, three clades differentiated by the genetic or DNA methylation state and one differentiating *G. barbadense* and Coker 315-11. Coker 315-11 is the most distant genotype both genetically and in DNA methylation to the other *G. hirsutum* genotypes. The dendrogram relationship pattern of the *G. hirsutum* genotypes (except Coker 315-11) differs within each genetic or DNA methylation clade, indicating that the genetic relation between each genotype is distinct from the DNA methylation relation.

The genetic and DNA methylation dendrograms were used to compare with the fibre length or strength-based dendrograms to assess whether DNA methylation was contributing more than the genetic component to fibre quality. There were no statistically significant relations between fibre lengths to the genetic or DNA methylation diversity. Weak but statistically significant positive correlation at p<0.05 level between fibre strength to the genetic and DNA methylation diversity was found, but the DNA methylation was no more correlated to fibre strength than the genetic component.

#### Visualizing diversity by Principal Component Analysis (PCA)

**Figure 6:**
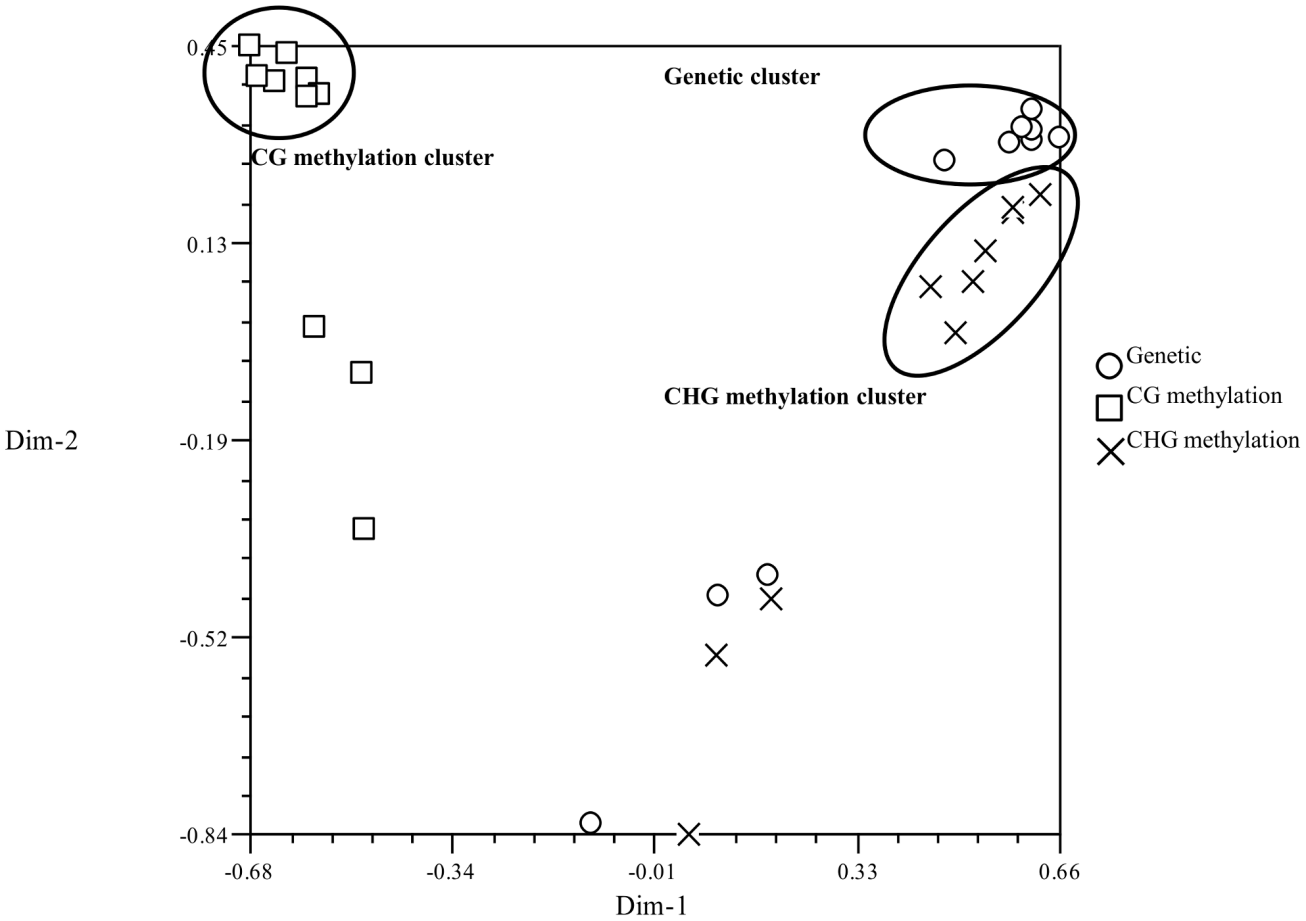
PCA of the ten genotypes for each enzyme combinations plotted in two dimensions. The spatial distance on the graph represents the genetic/DNA methylation relationship between each genotype. Genetic and DNA methylation relationship between the genotypes is more distant between the genetic and DNA methylation state forming genetic, CG methylation, CHG methylation clusters. The CHG methylation and genetic component are closely related, whereas the CG methylation is more distant. The *G. barbadense* genotypes (CPX12 and Sipima 280) and Coker 315-11 are outliers that are distant from the three clusters, genetically and epigenetically.

Principal component analysis (PCA) was performed to visualise the relative distance of each genotype, the genetic and DNA methylation state, using the similarity coefficient (Figure 6). The first and second dimension contributes 27.3% and 14.3% (cumulates to 41.6%), respectively, of the relationship in the multivariate space. CHG methylation was closer to the genetic similarity of *G. hirsutum* genotypes, and CG methylation was distant to both genetic and CHG methylation. Coker 315-11 and the two *G. barbadense* genotypes were distant from the other genotypes in both their genetic and methylation relationships.

Bootstrap analysis indicated that the divergence of Coker 315-11 and the two *G. barbadense* genotypes (CPX12, and Sipima 280) from the others are statistically significant (p<0.05) in their genetic relationship. Within the *G. hirsutum* only, the divergence of Coker 315-11 and other *G. hirsutum* genotypes was statistically significant for genetic, CG, and CHG methylation relationships (p-value of <0.05).

## Discussion

Epigenetic regulation is known to be involved in some traits in cotton [20]–[22], [33], [34], and has the potential to create phenotypic diversity that can improve agronomical performance. Spontaneous DNA methylation changes resulted in epigenomic divergence over as little as 30 generations in *Arabidopsis thaliana* [36], [37], suggesting that the 1–2 million years since allopolyploidization of cotton [44] would be sufficient to allow significant genetic, epigenetic, and phenotypic divergence between cotton species. We also found changes in absolute DNA methylation levels between various tissues, including fibre, but no significant difference was found between DNA methylation in leaf tissues across the ten genotypes representing a range of fibre length and strength. By contrast, MSAP analyses showed that DNA methylation diversity of the ten genotypes was higher than the genetic diversity. The higher level of DNA methylation diversity may change gene expression, adding to the higher phenotypic diversity that cannot be explained by the limited genetic diversity in cotton [41]–[43], [71], [72]. Our results demonstrate that epigenetic regulation is likely to be involved in cotton development and phenotypic diversity, and therefore has potential value for improving agronomic performance.

A unique feature of the MSAP method presented for the first time in this study is the inclusion of the enzyme, *BsiSI*, which is an isoschizomer of *Hpa*II and *Msp*I that recognises CCGG sequence, but is not methylation sensitive. With the aid of *Bsi*SI, the fully methylated sites containing both CG and CHG methylation have been classified as CC (also includes double-strand CHG methylation, because *Hpa*II cannot cleave CCGG methylated on the outer C on both strands), adding further sites that could not be assessed by the conventional MSAP method. More importantly, the use of *Bsi*SI allowed us to directly compare the genetic diversity to the DNA methylation diversity at the same CCGG site. The genetic diversity of *G. hirsutum* genotypes was very low, as expected in this species that has gone through a number of genetic bottlenecks during polyploidization, domestication and modern breeding [41]–[43], but the CG and CHG methylation diversity was always higher than the genetic diversity. Notably, the CHG methylation polymorphism was less frequent (thus less differentiated from the genetic polymorphisms) but occurred more randomly across the genotypes for each site, leading to high diversity and provides some evidence for possible small RNA mediated regulation of phenotypes. Consistent with this, CHG methylation can be guided and maintained by small RNAs, CMT3 and DRMs [73], and small RNAs have been shown to be involved in fibre development and altered by viral infection during fibre development in cotton [74]–[78].

Although no differences were observed for total DNA methylation level measured by HPLC, the pattern of DNA methylation, as determined by MSAP was different between some genotypes. HPLC was technically more accurate than MSAP in determining the total methylation level, probably due to technical errors of the MSAP method [67], [79]. However, while HPLC quantifies the methylated cytosine content of DNA, it cannot distinguish between the different methylation contexts. MSAP can measure CG and CHG methylation context changes, but generally under-estimates the methylation level as it: does not detect hypermethylated sites, and the dominant nature of the AFLP detects mixed methylated/non-methylated loci as non-methylated site. Despite this, the total methylation level estimated by MSAP (50%) was approximately double the HPLC measurement. Similar results were seen in *Brassica oleracea* where more than 3-times global methylation was detected by MSAP compared to HPLC [39]. The difference between HPLC and MSAP may partly be caused by the bias of assessing more methylated region of the genome (CCGG sites) in the MSAP method, where CG methylation is the most common context of DNA methylation [1], [2], whereas the HPLC method assesses the total methylated cytosines of the genome (including the non-methylated organellar genomes).

Higher DNA methylation diversity than the genetic diversity has also been demonstrated in another study where cotton plants from different geographic regions were sampled to monitor CG methylation patterns using conventional MSAP [45]. In this study of 20 *G. hirsutum* accessions grown in different geographical regions, the level of CG methylation polymorphism was 67%. This was somewhat higher than we observed in the eight *G. hirsutum* genotypes (59.2% CG polymorphism), although the reasons for this are unclear. Our study demonstrated that the DNA methylation diversity remains high even in cotton genotypes that were grown in the same environment over many generations. Similar results have been reported in other cultivated plants that suggest the involvement of epigenetic variation compensating for the lack of genetic variation [80], [81].

*G. hirsutum* (CSX4184, CSX5150, CSX6280, DP16, Namcala, Sicot 71, Sicot 75, and Coker 315) and *G. barbadense* (CPX12, and Sipima 280) total DNA methylation level determined by MSAP were 43% and 44.8% (CG and CHG, without CC methylation), respectively, and falls within the DNA methylation level range (16–60%) for other plant species [39], [48], [82], [83]. A large range in total methylation level in leaf DNA has been reported from other studies of *G. hirsutum* (19–37%), and the total methylation level measured in our study was about 6 percentage points higher than the upper level of these studies [20], [21], [33], [45]. The higher methylation level may result from the difference of technical errors, genotypes and/or environmental conditions used.

The level and context of DNA methylation of selected cotton genotypes and various tissues of Coker 315–11 were assessed using HPLC and MSAP. Higher methylation in mature tissues compared to developing tissues has been reported in other plant species [84]–[87], and this was also observed in cotton stems and roots, perhaps reflecting the accumulation of methylation over time. Care is needed in the interpretation of methylation changes between tissues as the number of plastids can vary depending on the tissue [88], and plastid DNA is generally not (or very lowly) methylated [89], [90], leading to underestimation of the total methylation level for plastid rich tissues (e.g. leaf). Isolation of nuclei is more accurate for quantifying DNA methylation level, but is technically difficult especially in fibre where limited amount of tissue was available (e.g. only small amount of outer and inner integument material can be obtained from each flower).

The significant increase of total DNA methylation in 35 dpa fibre compared to 0 dpa, 3 dpa ovules, stems and roots indicates a possible involvement of epigenetic regulation during fibre development. The relatively low DNA methylation level in 0 dpa ovules compared to the dissected outer and inner integuments suggests that nucellar tissues are hypomethylated, causing an overall decrease in ovule DNA methylation level. By 3 dpa, the nucellus appears smaller and the endosperm is undergoing rapid development [91]. The endosperm of Arabidopsis and rice is known to be hypomethylated [16], [19], which may also decrease the total DNA methylation level of 3 dpa ovules relative to other tissues.

There were no statistical differences between the total DNA methylation level between the outer integument and 35 dpa fibres, but MSAP analyses showed that there was considerable methylation polymorphism (97 loci). This contrasts strongly with the similarity of methylation level and fewer polymorphism seen between inner and outer integument (31). The polymorphism between 35 dpa fibre and outer integument may be associated with genes involved in fibre development, and the polymorphism between outer integument to inner integument may represent candidate loci involved in fibre initiation that are epigenetically regulated. However, as the outer integument consists of both fibre initials (about 30%) and epidermal cells, changes in methylation may not necessarily (only) be associated with fibre development. Nevertheless, the change in DNA methylation between fibre and other tissues hint that some genes may be epigenetically regulated for fibre development, supported by other studies that show potential involvement of small RNA directed DNA methylation [35], [76], [78]. Sequencing of differentially amplified fragments may provide further insight into the role of DNA methylation and gene expression during these fibre development stages.

DNA methylation changes during fibre development have shown the potential involvement of epigenetic regulation that may influence fibre quality. However, the DNA methylation pattern (of leaves) did not show any more correlation to fibre length and fibre strength, compared to the genetic pattern. The number of cotton genotypes that was assessed was too small to identify an association between DNA methylation and fibre quality. It is important to note that DNA methylation is only one level of multi-layered epigenetic regulation (such as histone modifications), and the DNA methylation diversity measured in this study may be underestimating the epigenetic diversity. Further work investigating DNA methylation and other epigenetic changes during different fibre developmental stages or fibre cells across multiple cultivars may provide an understanding of the epigenetic regulation of fibre traits. The high DNA methylation polymorphism may provide sufficient diversity for epigenome based breeding, even in crops with limited genetic diversity, with further investigation to link the polymorphisms to traits of interest.

## Supporting Information

Figure S1 Statistically significant differences are denoted by different letters. Fibre length and strength of the ten genotypes measured using HVI. Both graphs are arranged in ascending order. The *G. hirsutum* genotypes represent a range of fibre lengths and strengths, and *G. barbadense* represents longer and stronger fibre compared to *G. hirsutum*.
(TIF)

Figure S2 Dendrogram representing the relationship between tissues and CG/CHG methylation. “EH” represents *Eco*RI/*Hpa*II, and “EM” represents *Eco*RI/*Msp*I. The tissues are each represented by; 0 dpa = 0 dpa ovules, 3 dpa = 3 dpa ovules, Cot = Cotyledons, St = plantlet stems, RT = plantlet roots, ML = mature (fully expanded) leaf from mature plant, OI = 0 dpa ovule outer integument, II = 0 dpa ovule inner integument, and 35F = 35 dpa fibres. Mantel’s test supports the reliability of the dendrogram (r = 0.985). The error rate for each tissue comparison was determined by the number of absent peaks in either of the tissue in the *Eco*RI/*Bsi*SI data (i.e. all tissues should be genetically identical). Comparison of Outer integument-35 dpa fibre had 1.8% error rate, and Outer integument-Inner integument had 2.57% error rate.
(TIF)

Figure S3 Venn diagram representing the number of CG and CHG polymorphic fragments that are unique to each tissue and the number of polymorphic fragments that are shared between tissues. The 35 dpa fibre was unique with the highest numbers of specific polymorphism between the three tissues, for both CG and CHG methylation context.
(TIF)

Table S1 Error rate of HPLC method. The standard error of mean of different steps of the HPLC method was evaluated from six DNA extraction replicates, two sets of four DNA digest replicates, five HPLC run replicates (using the same DNA sample), and four day-to-day independent runs of standards. The %mdC and standard error of mean was calculated for each trial. The highest variation was observed in the day-to-day run with a standard error of mean at +/−0.54%.
(DOCX)

Table S2 List of oligonucleotides used for MSAP. Selective oligonucleotides with fluorescent labels indicated beside primer name (FAM, VIC, NED or PET).
(DOCX)

Table S3 Classification of methylation type for MSAP. Example of four types of methylation classification and the possible polymorphisms is represented by comparing two genotypes. “1” represents presence of bands and “0” represents absence of bands. Example of determining the methylation state is shown from “Cultivar A” and the polymorphism determined from comparing “Cultivar A” and “Cultivar B”.
(DOCX)

Table S4 Statistical validation of dendrogram and calculated error rate for MSAP. The r-value was determined for each constructed dendrogram (r-value >0.9 indicates good reliability of data). The average error rate per locus was calculated from the three biological replicates for *Eco*RI/*Bsi*SI, *Eco*RI/*Hpa*II, and *Eco*RI/*Msp*I from all genotypes.
(DOCX)

Table S5 Matrix correlation (Mantel’s test) between fibre quality and genetic/methylation relationship of cotton genotypes. When comparing the correlation coefficient between matrices with n = 10, coefficient above 0.282 is statistically significant at the 5% level and 0.445 at the 1% level (Lapointe & Legendre, 1992).
(DOCX)

Table S6 Percentage of polymorphisms identified in each enzyme combination. The percentage represents the number of polymorphic sites within the total number of sites analysed. The percentage polymorphism in the *Eco*RI/*Hpa*II and *Eco*RI/*Msp*I does not include the polymorphic sites identified in *Eco*RI/*Bsi*SI.
(DOCX)

## References

[1] Cokus SJ, Feng S, Zhang X, Chen Z, Merriman B, et al (2008) Shotgun bisulphite sequencing of the Arabidopsis genome reveals DNA methylation patterning. Nature 452: 215–219. 18278030 10.1038/nature06745 PMC2377394

[2] Lister R, O’Malley RC, Tonti-Filippini J, Gregory BD, Berry CC, et al (2008) Highly integrated single-base resolution maps of the epigenome in Arabidopsis. Cell 133: 523–536. 18423832 10.1016/j.cell.2008.03.029 PMC2723732

[3] Schöb H, Grossniklaus U (2006) The First High-Resolution DNA “Methylome”. Cell 126: 1025–1028. 16990127 10.1016/j.cell.2006.09.002

[4] Zhang M, Kimatu JN, Xu K, Liu B (2010) DNA cytosine methylation in plant development. Journal of Genetics and Genomics 37: 1–12. 20171573 10.1016/S1673-8527(09)60020-5

[5] Hauser M-T, Aufsatz W, Jonak C, Luschnig C (2011) Transgenerational epigenetic inheritance in plants. Biochimica et Biophysica Acta (BBA) - Gene Regulatory Mechanisms 1809: 459–468. 21515434 10.1016/j.bbagrm.2011.03.007 PMC4359895

[6] Henderson IR, Jacobsen SE (2007) Epigenetic inheritance in plants. Nature 447: 418–424. 17522675 10.1038/nature05917

[7] Chan SWL, Henderson IR, Jacobsen SE (2005) Gardening the genome: DNA methylation in Arabidopsis thaliana. Nature reviews Genetics 6: 351–360. 10.1038/nrg1601 15861207

[8] Vongs A, Kakutani T, Martienssen RA, Richards EJ (1993) Arabidopsis thaliana DNA methylation mutants. Science (New York, NY) 260: 1926–1928. 10.1126/science.8316832 8316832

[9] Kakutani T, Kato M, Kinoshita T, Miura A (2004) Control of development and transposon movement by DNA methylation in Arabidopsis thaliana. Cold Spring Harbor Symposia on Quantitative Biology 69: 139–143. 16117643 10.1101/sqb.2004.69.139

[10] Finnegan EJ, Peacock WJ, Dennis ES (1996) Reduced DNA methylation in Arabidopsis thaliana results in abnormal plant development. 93: 8449–8454. 10.1073/pnas.93.16.8449 PMC38691 8710891

[11] Lu Y, Rong T, Cao M (2008) Analysis of DNA methylation in different maize tissues. Journal of Genetics 35: 41–48. 10.1016/S1673-8527(08)60006-5 18222408

[12] Xiong LZ, Xu CG, Maroof MAS, Zhang QF (1999) Patterns of cytosine methylation in an elite rice hybrid and its parental lines, detected by a methylation-sensitive amplification polymorphism technique. Molecular and General Genetics 261: 439–446. 10323223 10.1007/s004380050986

[13] Zhang M, Xu C, von Wettstein D, Liu B (2011) Tissue-Specific Differences in Cytosine Methylation and their Association with Differential Gene Expression in Sorghum bicolar. Plant physiology. 10.1104/pp.111.176842 PMC3149958 21632971

[14] Ruiz-Garcia L, Cervera MT, Martinez-Zapater JM (2005) DNA methylation increases throughout Arabidopsis development. Planta 222: 301–306. 15968513 10.1007/s00425-005-1524-6

[15] Messeguer R, Ganal MW, Steffens JC, Tanksley SD (1991) Characterization of the level, target sites and inheritance of cytosine methylation in tomato nuclear-DNA. Plant Molecular Biology 16: 753–770. 1859863 10.1007/BF00015069

[16] Zemach A, Kim MY, Silva P, Rodrigues JA, Dotson B, et al (2010) Local DNA hypomethylation activates genes in rice endosperm. Proceedings of the National Academy of Sciences of the United States of America 107: 18729–18734. 20937895 10.1073/pnas.1009695107 PMC2972920

[17] Zhang MS, Yan HY, Zhao N, Lin XY, Pang JS, et al (2007) Endosperm-specific hypomethylation, and meiotic inheritance and variation of DNA methylation level and pattern in sorghum (Sorghum bicolor L.) inter-strain hybrids. Theoretical and Applied Genetics 115: 195–207. 17486309 10.1007/s00122-007-0555-8

[18] Lauria M, Rupe M, Guo M, Kranz E, Pirona R, et al (2004) Extensive Maternal DNA Hypomethylation in the Endosperm of Zea mays. Society 16: 510–522. 10.1105/tpc.017780 PMC341920 14729913

[19] Hsieh T-f (2011) Genome-Wide Demethylation of Arabidopsis endosperm. Science 1451. 10.1126/science.1172417 PMC4044190 19520962

[20] Zhao Y, Yu S, Xing C, Fan S, Song M (2008) Analysis of DNA methylation in cotton hybrids and their parents. Molecular Biology 42: 169–178. 18610827

[21] Cao DH, Gao X, Liu J, Kimatu JN, Geng SJ, et al (2011) Methylation sensitive amplified polymorphism (MSAP) reveals that alkali stress triggers more DNA hypomethylation levels in cotton (Gossypium hirsutum L.) roots than salt stress. African Journal of Biotechnology 10: 18971–18980.

[22] Zhao Y-l, Yu S-x, Ye W-w, Wang H-m, Wang J-j, et al (2010) Study on DNA Cytosine Methylation of Cotton (Gossypium hirsutum L.) Genome and Its Implication for Salt Tolerance. Agricultural Sciences in China 9: 783–791.

[23] Jacobsen SE, Sakai H, Finnegan EJ, Cao X, Meyerowitz EM (2000) Ectopic hypermethylation of flower-specific genes in Arabidopsis. Current Biology 10: 179–186. 10704409 10.1016/s0960-9822(00)00324-9

[24] Kakutani T, Jeddeloh JA, Richards EJ (1995) Characterization of an Arabidopsis thaliana DNA hypomethylation mutant. Nucleic acids research 23: 130–137. 7870578 10.1093/nar/23.1.130 PMC306640

[25] Bossdorf O, Arcuri D, Richards CL, Pigliucci M (2010) Experimental alteration of DNA methylation affects the phenotypic plasticity of ecologically relevant traits in Arabidopsis thaliana. Evolutionary Ecology 24: 541–553.

[26] Amoah S, Kurup S, Lopez CMR, Welham SJ, Powers SJ, et al.. (2012) A Hypomethylated population of Brassica rapa for forward and reverse Epi-genetics. Bmc Plant Biology 12. 10.1186/1471-2229-12-193 PMC3507869 23082790

[27] Manning K, Tör M, Poole M, Hong Y, Thompson AJ, et al (2006) A naturally occurring epigenetic mutation in a gene encoding an SBP-box transcription factor inhibits tomato fruit ripening. Nature genetics 38: 948–952. 16832354 10.1038/ng1841

[28] Martin A, Troadec C, Boualem A, Rajab M, Fernandez R, et al (2009) A transposon-induced epigenetic change leads to sex determination in melon. Nature 461: 1135–1138. 19847267 10.1038/nature08498

[29] Miura K, Agetsuma M, Kitano H, Yoshimura A, Matsuoka M, et al (2009) A metastable DWARF1 epigenetic mutant affecting plant stature in rice. Proceedings of the National Academy of Sciences 106: 11218–11223. 10.1073/pnas.0901942106 PMC2708680 19541604

[30] Hauben M, Haesendonckx B, Standaert E, Van Der Kelen K, Azmi A, et al (2009) Energy use efficiency is characterized by an epigenetic component that can be directed through artificial selection to increase yield. Proceedings of the National Academy of Sciences of the United States of America 106: 20109–20114. 19897729 10.1073/pnas.0908755106 PMC2774259

[31] Zhong S, Fei Z, Chen Y-R, Zheng Y, Huang M, et al (2013) Single-base resolution methylomes of tomato fruit development reveal epigenome modifications associated with ripening. Nat Biotech 31: 154–159. 10.1038/nbt.2462 23354102

[32] Finnegan EJ, Genger RK, Kovac K, Peacock WJ, Dennis ES (1998) DNA methylation and the promotion of flowering by vernalization. Proceedings of the National Academy of Sciences of the United States of America 95: 5824–5829. 9576969 10.1073/pnas.95.10.5824 PMC20464

[33] Li TC, Fan HH, Li ZP, Wei J, Cai YP, et al (2011) Effect of different light quality on DNA methylation variation for brown cotton (Gossypium hirstum). African Journal of Biotechnology 10: 6220–6226.

[34] Li X-L, Lin Z-X, Nie Y-C, Guo X-P, Zhang X-L (2009) Methylation-Sensitive Amplification Polymorphism of Epigenetic Changes in Cotton Under Salt Stress. Acta Agronomica Sinica 35: 588–596.

[35] Jin X, Pang Y, Jia F, Xiao G, Li Q, et al (2013) A Potential Role for CHH DNA Methylation in Cotton Fiber Growth Patterns. PLoS ONE 8: e60547. 23593241 10.1371/journal.pone.0060547 PMC3625195

[36] Becker C, Hagmann J, Muller J, Koenig D, Stegle O, et al (2011) Spontaneous epigenetic variation in the Arabidopsis thaliana methylome. Nature 480: 245–249. 22057020 10.1038/nature10555

[37] Schmitz RJ, Schultz MD, Lewsey MG, O’Malley RC, Urich MA, et al (2011) Transgenerational Epigenetic Instability Is a Source of Novel Methylation Variants. Science 334: 369–373. 21921155 10.1126/science.1212959 PMC3210014

[38] Herrera CM, Bazaga P (2010) Epigenetic differentiation and relationship to adaptive genetic divergence in discrete populations of the violet Viola cazorlensis. New Phytologist 187: 867–876. 20497347 10.1111/j.1469-8137.2010.03298.x

[39] Salmon A, Clotault J, Jenczewski E, Chable V, Manzanares-Dauleux MJ (2008) Brassica oleracea displays a high level of DNA methylation polymorphism. Plant Science 174: 61–70.

[40] Campbell BT, Williams VE, Park W (2009) Using molecular markers and field performance data to characterize the Pee Dee cotton germplasm resources. Euphytica 169: 285–301.

[41] Abdalla AM, Reddy OUK, El-Zik KM, Pepper AE (2001) Genetic diversity and relationships of diploid and tetraploid cottons revealed using AFLP. TAG Theoretical and Applied Genetics 102: 222–229.

[42] Wendel JF, Brubaker CL, Percival AE (1992) Genetic diversity in gossypium-hirsutum and the origin of upland cotton. American Journal of Botany 79: 1291–1310.

[43] Iqbal MJ, Aziz N, Saeed NA, Zafar Y, Malik KA (1997) Genetic diversity evaluation of some elite cotton varieties by RAPD analysis. Theoretical and Applied Genetics 94: 139–144. 19352756 10.1007/s001220050392

[44] Wendel JF, Cronn RC (2003) Polyploidy and the evolutionary history of cotton. Advances in Agronomy 78: 139–186.

[45] Keyte AL, Percifield R, Liu B, Wendel JF (2006) Intraspecific DNA methylation polymorphism in cotton (Gossypium hirsutum L.). The Journal of heredity 97: 444–450. 16987937 10.1093/jhered/esl023

[46] Lovell D, Wu Y, White R, Machado A, Llewellyn DJ, et al (2007) Phenotyping cotton ovule fibre initiation with spatial statistics. Australian Journal of Botany 55: 608–608.

[47] Lee JJ, Woodward AW, Chen ZJ (2007) Gene expression changes and early events in cotton fibre development. Annals of botany 100: 1391–1401. 17905721 10.1093/aob/mcm232 PMC2759220

[48] Shaked H, Kashkush K, Ozkan H, Feldman M, Levy AA (2001) Sequence elimination and cytosine methylation are rapid and reproducible responses of the genome to wide hybridization and allopolyploidy in wheat. Plant Cell 13: 1749–1759. 11487690 10.1105/TPC.010083 PMC139131

[49] Kashkush K, Feldman M, Levy AA (2002) Gene loss, silencing and activation in a newly synthesized wheat allotetraploid. Genetics 160: 1651–1659. 11973318 10.1093/genetics/160.4.1651 PMC1462064

[50] Wang J, Tian L, Madlung A, Lee H-S, Chen M, et al (2004) Stochastic and epigenetic changes of gene expression in Arabidopsis polyploids. Genetics 167: 1961–1973. 15342533 10.1534/genetics.104.027896 PMC1471021

[51] Xu YH, Zhong L, Wu XM, Fang XP, Wang JB (2009) Rapid alterations of gene expression and cytosine methylation in newly synthesized Brassica napus allopolyploids. Planta 229: 471–483. 18998158 10.1007/s00425-008-0844-8

[52] Mansoor S, Paterson AH (2012) Genomes for jeans: cotton genomics for engineering superior fiber. Trends in Biotechnology 30: 521–527. 22831638 10.1016/j.tibtech.2012.06.003

[53] Isbell RF, editor (1996) The Australian soil classification: CSIRO publishing.

[54] Bedon F, Ziolkowski L, Osabe K, Venables I, Machado A, et al (2013) Separation of integument and nucellar tissues from cotton ovules (*Gossypium hirsutum* L.) for both high- and low-throughput molecular applications. BioTechniques 54: 44–46. 10.2144/000113969 23237485

[55] Quinlivan EP, Gregory JF III (2008) DNA digestion to deoxyribonucleoside: A simplified one-step procedure. Analytical Biochemistry 373: 383–385. 18028864 10.1016/j.ab.2007.09.031 PMC2239294

[56] Johnston JW, Harding K, Bremner DH, Souch G, Green J, et al (2005) HPLC analysis of plant DNA methylation: a study of critical methodological factors. Plant Physiology and Biochemistry 43: 844–853. 16289949 10.1016/j.plaphy.2005.07.015

[57] Vos P, Hogers R, Bleeker M, Reijans M, Vandelee T, et al (1995) AFLP - a new technique for DNA-fingerprinting. Nucleic Acids Research 23: 4407–4414. 7501463 10.1093/nar/23.21.4407 PMC307397

[58] Rohlf FJ (2008) NTSYSpc: Numerical Taxonomy System, ver. 2.21. Setauket, NY: Exeter Publishing, Ltd.

[59] Halldén C, Nilsson NO, Rading IM, Säll T (1994) Evaluation of RFLP and RAPD markers in a comparison of Brassica napus breeding lines. Theoretical and Applied Genetics 88: 123–128. 24185892 10.1007/BF00222404

[60] Dice LR (1945) Measures of the amount of ecologic association between species. Ecology 26: 297–302.

[61] Jaccard P (1901) Etude comparative de la distribution florale dans une portion des Alpes et du Jura. Bulletin de la Société vaudoise des Sciences Naturelles 37: 547–579.

[62] Laurentin H (2009) Data analysis for molecular characterization of plant genetic resources. Genetic Resources and Crop Evolution 56: 277–292.

[63] Garcia-Vallve S, Palau J, Romeu A (1999) Horizontal gene transfer in glycosyl hydrolases inferred from codon usage in Escherichia coli and Bacillus subtilis. Molecular Biology and Evolution 16: 1125–1134. 10486968 10.1093/oxfordjournals.molbev.a026203

[64] Stricker D (2008) BrightStat.com: Free statistics online. Comput Methods Prog Biomed 92: 135–143. 10.1016/j.cmpb.2008.06.010 18653259

[65] Hedges SB (1992) The number of replications needed for accurate estimation of the bootstrap-p value in phylogenetic studies. Molecular Biology and Evolution 9: 366–369. 1560769 10.1093/oxfordjournals.molbev.a040725

[66] Nelson R (1995) WinBoot: A program for performing bootstrap analysis of binary data to determine the confidence limits of UPGMA-based dendrograms. IRRI Discussion Paper Series No 14.

[67] Pompanon F, Bonin A, Bellemain E, Taberlet P (2005) Genotyping errors: causes, consequences and solutions. Nat Rev Genet 6: 847–846. 16304600 10.1038/nrg1707

[68] Walford S-A, Wu Y, Llewellyn DJ, Dennis ES (2011) GhMYB25-like: a key factor in early cotton fibre development. The Plant journal : for cell and molecular biology 65: 785–797. 21235650 10.1111/j.1365-313X.2010.04464.x

[69] Gore UR (1932) Development of the female gametophyte and embryo in cotton. American Journal of Botany 19: 795–807.

[70] Lintilha PM, Jensen WA (1974) Differentiation, organogenesis, and tectonics of cell-wall orientation.1. Preliminary observations on development of ovule in cotton. American Journal of Botany 61: 129–134.

[71] Brubaker CL, Bourland FM, Wendel JF (1999) The origin and domestication of cotton. In: Smith W, editor. Cotton: origin, history, technology, and production. New York: John Wiley & Sons. 3–31.

[72] Applequist WL, Cronn R, Wendel JF (2001) Comparative development of fiber in wild and cultivated cotton. Evolution & Development 3: 3–17. 11256432 10.1046/j.1525-142x.2001.00079.x

[73] Cao X, Aufsatz W, Zilberman D, Mette MF, Huang MS, et al (2003) Role of the DRM and CMT3 Methyltransferases in RNA-Directed DNA Methylation. Current 13: 2212–2217. 10.1016/j.cub.2003.11.052 14680640

[74] Romanel E, Silva TF, Correa RL, Farinelli L, Hawkins JS, et al (2012) Global alteration of microRNAs and transposon-derived small RNAs in cotton (Gossypium hirsutum) during Cotton leafroll dwarf polerovirus (CLRDV) infection. Plant Molecular Biology 80: 443–460. 22987114 10.1007/s11103-012-9959-1

[75] Kwak P, Wang Q, Chen X, Qiu C, Yang Z (2009) Enrichment of a set of microRNAs during the cotton fiber development. BMC Genomics 10: 457. 19788742 10.1186/1471-2164-10-457 PMC2760587

[76] Wang Z-M, Xue W, Dong C-J, Jin L-G, Bian S-M, et al.. (2011) A Comparative miRNAome Analysis Reveals Seven Fiber Initiation-Related and 36 Novel miRNAs in Developing Cotton Ovules. Molecular plant. 10.1093/mp/ssr094 22138860

[77] Pang MX, Xing CZ, Adams N, Rodriguez-Uribe L, Hughs SE, et al (2011) Comparative expression of miRNA genes and miRNA-based AFLP marker analysis in cultivated tetraploid cottons. Journal of Plant Physiology 168: 824–830. 21134704 10.1016/j.jplph.2010.10.006

[78] Li Q, Jin X, Zhu YX (2012) Identification and Analyses of miRNA Genes in Allotetraploid Gossypium hirsutum Fiber Cells Based on the Sequenced Diploid G-raimondii Genome. Journal of Genetics and Genomics 39: 351–360. 22835981 10.1016/j.jgg.2012.04.008

[79] Meudt HM, Clarke AC (2007) Almost forgotten or latest practice? AFLP applications, analyses and advances. Trends in Plant Science 12: 106–117. 17303467 10.1016/j.tplants.2007.02.001

[80] Fang J, Song C, Zheng Y, Qiao Y, Zhang Z, et al (2008) Variation in cytosine methylation in Clementine mandarin cultivars. Journal of Horticultural Science & Biotechnology 83: 833–839.

[81] Fang JG, Song CN, Qian JL, Zhang XY, Shangguan LF, et al (2010) Variation of cytosine methylation in 57 sweet orange cultivars. Acta Physiologiae Plantarum 32: 1023–1030.

[82] Ashikawa I (2001) Surveying CpG methylation at 5′-CCGG in the genomes of rice cultivars. Plant Molecular Biology 45: 31–39. 11247604 10.1023/a:1006457321781

[83] Takata M, Kishima Y, Sano Y (2005) DNA methylation polymorphisms in rice and wild rice strains: Detection of epigenetic markers. Breeding Science 55: 57–63.

[84] Sakowicz T, Olszewska MJ, Luchniak P, Kazmierczak J (1998) Tissue-specific DNA methylation in Haemanthus katharinae Bak. (amaryllidaceae). Acta Societatis Botanicorum Poloniae 67: 175–180.

[85] Kazmierczak J (1998) Effect of DNA methylation on potential transcriptional activity in different tissues and organs of Vicia faba ssp. minor. Folia Histochemica Et Cytobiologica 36: 45–49. 9527024

[86] Palmgren G, Mattsson O, Okkels FT (1991) Specific levels of dna methylation in various tissues, cell-lines, and cell-types of daucus-carota. Plant Physiology 95: 174–178. 16667947 10.1104/pp.95.1.174 PMC1077502

[87] Jia F, Fu Y, Liu W, Du Z, Zhao Y (2011) Quantitative determination of DNA Methylation in tobacco leaves by HPLC. Journal of Agricultural Research 6: 1545–1548.

[88] Scott NS, Tymms MJ, Possingham JV (1984) Plastid-DNA Levels In The Different Tissues Of Potato. Planta 161: 12–19. 24253550 10.1007/BF00951454

[89] Marano MR, Carrillo N (1991) Chromoplast formation during tomato fruit ripening - no evidence for plastid dna methylation. Plant Molecular Biology 16: 11–19. 1653626 10.1007/BF00017913

[90] Fojtova M, Kovarik A, Matyasek R (2001) Cytosine methylation of plastid genome in higher plants. Fact or artefact? Plant Science 160: 585–593. 11448733 10.1016/s0168-9452(00)00411-8

[91] Schulz P, Jensen WA (1977) Cotton Embryogenesis: The Early Development of the Free Nuclear Endosperm. American Journal of Botany 64: 384–394.

