# Supplementary figures and images for: Genetic and DNA Methylation Changes in Cotton (Gossypium) Genotypes and Tissues
Source: PLoS One. 2014 Jan 20;9(1):e86049. doi: 10.1371/journal.pone.0086049 (PMC3896429; doi:10.1371/journal.pone.0086049)

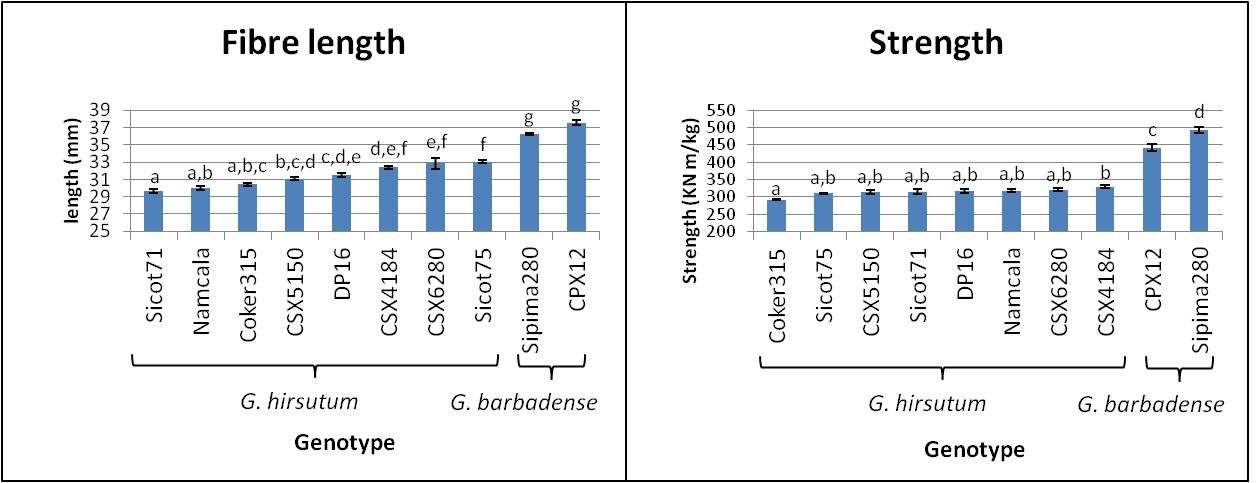

Supplement: Figure S1 — Statistically significant differences are denoted by different letters. Fibre length and strength of the ten genotypes measured using HVI. Both graphs are arranged in ascending order. The G. hirsutum genotypes represent a range of fibre lengths and strengths, and G. barbadense represents longer and stronger fibre compared to G. hirsutum. (TIF) [file pone.0086049.s001.tif]

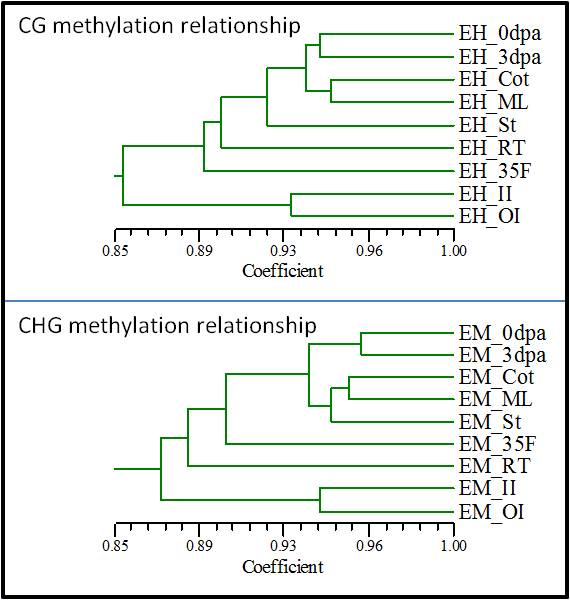

Supplement: Figure S2 — Dendrogram representing the relationship between tissues and CG/CHG methylation. “EH” represents EcoRI/HpaII, and “EM” represents EcoRI/MspI. The tissues are each represented by; 0 dpa = 0 dpa ovules, 3 dpa = 3 dpa ovules, Cot = Cotyledons, St = plantlet stems, RT = plantlet roots, ML = mature (fully expanded) leaf from mature plant, OI = 0 dpa ovule outer integument, II = 0 dpa ovule inner integument, and 35F = 35 dpa fibres. Mantel’s test supports the reliability of the dendrogram (r = 0.985). The error rate for each tissue comparison was determined by the number of absent peaks in either of the tissue in the EcoRI/BsiSI data (i.e. all tissues should be genetically identical). Comparison of Outer integument-35 dpa fibre had 1.8% error rate, and Outer integument-Inner integument had 2.57% error rate. (TIF) [file pone.0086049.s002.tif]

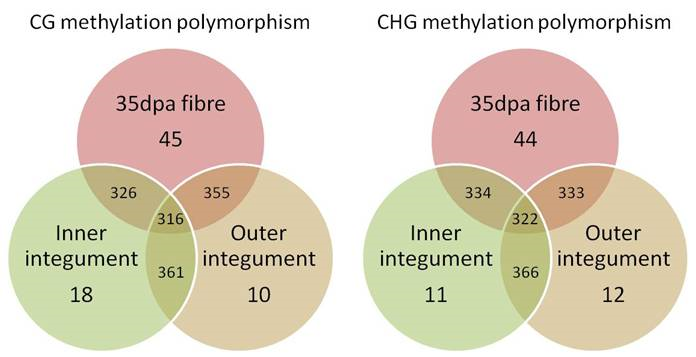

Supplement: Figure S3 — Venn diagram representing the number of CG and CHG polymorphic fragments that are unique to each tissue and the number of polymorphic fragments that are shared between tissues. The 35 dpa fibre was unique with the highest numbers of specific polymorphism between the three tissues, for both CG and CHG methylation context. (TIF) [file pone.0086049.s003.tif]
