# Supplementary material for: Genetic and DNA Methylation Changes in Cotton (Gossypium) Genotypes and Tissues
Source: PLoS One. 2014 Jan 20;9(1):e86049. doi: 10.1371/journal.pone.0086049 (PMC3896429; doi:10.1371/journal.pone.0086049)
Supplement: Table S1 — Error rate of HPLC method. The standard error of mean of different steps of the HPLC method was evaluated from six DNA extraction replicates, two sets of four DNA digest replicates, five HPLC run replicates (using the same DNA sample), and four day-to-day independent runs of standards. The %mdC and standard error of mean was calculated for each trial. The highest variation was observed in the day-to-day run with a standard error of mean at +/−0.54%. (DOCX) [file pone.0086049.s004.docx]

Table S1. Error rate of HPLC method.

| Replicates | Standard error of mean (%) |
| --- | --- |
| DNA extraction | 0.13 |
| DNA digest | 0.11 |
| HPLC run | 0.05 |
| Day-to-day | 0.54 |

The standard error of mean of different steps of the HPLC method was evaluated from six DNA extraction replicates, two sets of four DNA digest replicates, five HPLC run replicates (using the same DNA sample), and four day-to-day independent runs of standards. The %mdC and standard error of mean was calculated for each trial. The highest variation was observed in the day-to-day run with a standard error of mean at +/-0.54%.
