# Supplementary material for: Genetic and DNA Methylation Changes in Cotton (Gossypium) Genotypes and Tissues
Source: PLoS One. 2014 Jan 20;9(1):e86049. doi: 10.1371/journal.pone.0086049 (PMC3896429; doi:10.1371/journal.pone.0086049)
Supplement: Table S2 — List of oligonucleotides used for MSAP. Selective oligonucleotides with fluorescent labels indicated beside primer name (FAM, VIC, NED or PET). (DOCX) [file pone.0086049.s005.docx]

Table S2. List of oligonucleotides used for MSAP.

| Use | Primer name | Sequence 5'->3' |
| --- | --- | --- |
| Adapters | EcoRI-FWD | CTCGTAGACTGCGTACC |
|  | EcoRI-REV | AATTGGTACGCAGTC |
|  | HpaII/MspI-FWD | GACGATGAGTCTAGAA |
|  | HpaII/MspI-REV | CGTTCTAGACTCATC |
|  | Preselective primers |  |
|  | EcoRI +A | GACTGCGTACCAATTCA |
|  | HpaII/MspI +T | GATGAGTCTAGAACGGT |
| Selective primers | EcoRI +AAG (FAM) | GACTGCGTACCAATTCAAG |
|  | EcoRI +ACA (VIC) | GACTGCGTACCAATTCACA |
|  | EcoRI +ACC (NED) | GACTGCGTACCAATTCACC |
|  | EcoRI +ACG (PET) | GACTGCGTACCAATTCACG |
|  | EcoRI +ACT (FAM) | GACTGCGTACCAATTCACT |
|  | EcoRI +AGT (VIC) | GACTGCGTACCAATTCAGT |
|  | EcoRI +AGC (NED) | GACTGCGTACCAATTCAGC |
|  | EcoRI AGG (PET) | GACTGCGTACCAATTCAGG |
|  | HpaII/MspI +TCC | GATGAGTCTAGAACGGTCC |
|  | HpaII/MspI +TAA | GATGAGTCTAGAACGGTAA |
|  | HpaII/MspI +TAG | GATGAGTCTAGAACGGTAG |
|  | HpaII/MspI +TAC | GATGAGTCTAGAACGGTAC |
|  | HpaII/MspI +TGA | GATGAGTCTAGAACGGTGA |
|  | HpaII/MspI +TGG | GATGAGTCTAGAACGGTGG |
|  | HpaII/MspI +TGC | GATGAGTCTAGAACGGTGC |

Selective oligonucleotides with fluorescent labels indicated beside primer name (FAM, VIC, NED or PET).
