# Supplementary material for: Genetic and DNA Methylation Changes in Cotton (Gossypium) Genotypes and Tissues
Source: PLoS One. 2014 Jan 20;9(1):e86049. doi: 10.1371/journal.pone.0086049 (PMC3896429; doi:10.1371/journal.pone.0086049)
Supplement: Table S3 — Classification of methylation type for MSAP. Example of four types of methylation classification and the possible polymorphisms is represented by comparing two genotypes. “1” represents presence of bands and “0” represents absence of bands. Example of determining the methylation state is shown from “Cultivar A” and the polymorphism determined from comparing “Cultivar A” and “Cultivar B”. (DOCX) [file pone.0086049.s006.docx]

Table S3. Classification of methylation type for MSAP.

| Classification of sites | | | | | | | | |
| --- | --- | --- | --- | --- | --- | --- | --- | --- |
|  | Cultivar A | | |  | Cultivar B | | |  |
|  | BsiSI | HpaII | MspI | Methylation | BsiSI | HpaII | MspI | Polymorphism |
| Site 1 | 1 | 1 | 1 | Non-methylated | 0 | 0 | 0 | Genetic polymorphism |
| Site 2 | 1 | 1 | 0 | CHG methylation | 1 | 1 | 1 | CHG methylation polymorphism |
| Site 3 | 1 | 0 | 1 | CG methylation | 1 | 1 | 1 | CG methylation polymorphism |
| Si*te* 4 | 1 | 0 | 0 | CC methylation | Change in CC methylation will lead to either/both CHG/CG methylation polymorphism | | | |

Example of four types of methylation classification and the possible polymorphisms is represented by comparing two genotypes. “1” represents presence of bands and “0” represents absence of bands. Example of determining the methylation state is shown from “Cultivar A” and the polymorphism determined from comparing “Cultivar A” and “Cultivar B”.
