# Supplementary material for: Genetic and DNA Methylation Changes in Cotton (Gossypium) Genotypes and Tissues
Source: PLoS One. 2014 Jan 20;9(1):e86049. doi: 10.1371/journal.pone.0086049 (PMC3896429; doi:10.1371/journal.pone.0086049)
Supplement: Table S4 — Statistical validation of dendrogram and calculated error rate for MSAP. The r-value was determined for each constructed dendrogram (r-value >0.9 indicates good reliability of data). The average error rate per locus was calculated from the three biological replicates for EcoRI/BsiSI, EcoRI/HpaII, and EcoRI/MspI from all genotypes. (DOCX) [file pone.0086049.s007.docx]

Table S4. Statistical validation of dendrogram and calculated error rate for MSAP.

|  | Genotype dendrograms (r-value) | | MSAP error rate (%) |
| --- | --- | --- | --- |
| EcoRI/BsiSI | | 0.95 | 3.8 |
| EcoRI/HpaII | | 0.92 | 4.4 |
| EcoRI/MspI | | 0.94 | 6.1 |

The r-value was determined for each constructed dendrogram (r-value >0.9 indicates good reliability of data). The average error rate per locus was calculated from the three biological replicates for *Eco*RI/*Bsi*SI, *Eco*RI/*Hpa*II, and *Eco*RI/*Msp*I from all genotypes.
