# Supplementary material for: Genetic and DNA Methylation Changes in Cotton (Gossypium) Genotypes and Tissues
Source: PLoS One. 2014 Jan 20;9(1):e86049. doi: 10.1371/journal.pone.0086049 (PMC3896429; doi:10.1371/journal.pone.0086049)
Supplement: Table S5 — Matrix correlation (Mantel’s test) between fibre quality and genetic/methylation relationship of cotton genotypes. When comparing the correlation coefficient between matrices with n = 10, coefficient above 0.282 is statistically significant at the 5% level and 0.445 at the 1% level (Lapointe & Legendre, 1992). (DOCX) [file pone.0086049.s008.docx]

Table S5. Matrix correlation (Mantel’s test) between fibre quality and genetic/methylation relationship of cotton genotypes.

|  | Length | Strength |
| --- | --- | --- |
| EcoRI/BsiSI | 0.12 | 0.40 |
| EcoRI/HpaII | 0.14 | 0.39 |
| EcoRI/MspI | 0.09 | 0.39 |

When comparing the correlation coefficient between matrices with n=10, coefficient above 0.282 is statistically significant at the 5% level and 0.445 at the 1% level ([Lapointe & Legendre, 1992](#_ENREF_40)).
