# Supplementary material for: Genetic and DNA Methylation Changes in Cotton (Gossypium) Genotypes and Tissues
Source: PLoS One. 2014 Jan 20;9(1):e86049. doi: 10.1371/journal.pone.0086049 (PMC3896429; doi:10.1371/journal.pone.0086049)
Supplement: Table S6 — Percentage of polymorphisms identified in each enzyme combination. The percentage represents the number of polymorphic sites within the total number of sites analysed. The percentage polymorphism in the EcoRI/HpaII and EcoRI/MspI does not include the polymorphic sites identified in EcoRI/BsiSI. (DOCX) [file pone.0086049.s009.docx]

Table S 6. Percentage of polymorphisms identified in each enzyme combination.

|  | Percentage polymorphism | |
| --- | --- | --- |
| Enzyme combination | Ten genotypes (*G. hirsutum* + *G. barbadense*) – 1120 bands | *G. hirsutum* only – 1084 bands |
| EcoRI/BsiSI | 36.16 | 21.77 |
| EcoRI/HpaII | 60.45 | 59.23 |
| EcoRI/MspI | 36.25 | 31.18 |

The percentage represents the number of polymorphic sites within the total number of sites analysed. The percentage polymorphism in the *Eco*RI/*Hpa*II and *Eco*RI/*Msp*I does not include the polymorphic sites identified in *Eco*RI/*Bsi*SI.
